# The Use of DNA Markers in Rice Breeding for Blast Resistance and Submergence Tolerance as a Weed Control Factor

**DOI:** 10.3390/plants14121815

**Published:** 2025-06-13

**Authors:** Elena Dubina, Pavel Kostylev, Yulia Makukha, Margarita Ruban

**Affiliations:** 1 Federal Scientific Rice Centre, Belozerny, 3, Krasnodar 350921, Russia; lenakrug1@rambler.ru (E.D.); makyxa69@mail.ru (Y.M.); margaritaruban8@gmail.com (M.R.); 2 Department of Genetics, Breeding and Seed Production, Faculty of Agronomy and Ecology, Kuban State Agrarian University Named After I.T. Trubilin, 13 Kalinin Str., Krasnodar 350044, Russia; 3 Department of Biotechnology, Biochemistry and Biophysics, Faculty of Food Production and Biotechnology, Kuban State Agrarian University Named After I.T. Trubilin, 13 Kalinin Str., Krasnodar 350044, Russia; 4 Agricultural Research Center “Donskoy”, Nauchny Gorodok, 3, Zernograd 347740, Russia

**Keywords:** rice, varieties, blast resistance genes, submergence tolerance genes, DNA marking technologies

## Abstract

Diseases and weeds occupy a leading place among the factors limiting the yield of agricultural crops, including rice. These factors can be overcome through the use of chemical protective agents, as well as through the creation and introduction into agricultural production of rice varieties resistant to these stressors. The use of DNA marking technologies for target genes of economically valuable traits in the creation of promising varieties allows not only for the identification of genes but also the monitoring of their transmission during crosses and the selection of breeding-valuable genotypes with genes of interest. In addition, this ensures a reduction in the volume of breeding nurseries, as well as time and material costs during variety modeling, and rapid rotation of new high-yield varieties with specified characteristics. We have selected effective marker systems based on the use of DNA marking technologies for target genes for resistance to blast (*Pi*) and submergence tolerance (*Sub1A*). These systems allow for precise targeted selection of hybrid plants with these genes in the breeding process. In addition, we have automated the detection of transferred *Pi-ta* and *Pi-b* genes, which greatly relieves the DNA analysis during mass screening of breeding material. The final result of this work is the created rice varieties Al’yans, Lenaris and Kapitan with the *Pi-ta* blast resistance gene and the Pirouette rice variety with the *Pi-1*, *Pi-2*, and *Pi-33* genes. These varieties exceed the standards by 0.64–2.2 t/ha, and their involvement in production makes it possible to obtain additional products by increasing yields in the amount of about RUB 80 thousand/ha.

## 1. Introduction

Rice (*Oryza sativa* L.) is one of the three most in-demand cereal crops grown annually worldwide. In 2024, the area for this crop in the Russian Federation amounted to 206 thousand hectares. The Krasnodar region is the largest rice-growing area in Russia, accounting for more than 60% of the acreage in the country. In 2024, 117.4 thousand hectares were sown with this crop in the region. In the Rostov region, rice was grown on an area of 14.6 thousand hectares. Diseases, as well as weeds, are limiting factors that prevent high yields of rice and reduce crop productivity. According to the degree of harmfulness to rice, blast disease (the causative agent is the imperfect fungus *Pyricularia oryzae* Cav. (*Magnaporthe grisea* (Herbert) Barr)) takes first place.

The pathogen affects all above-ground organs of the plant and represents a danger during the entire vegetative period of the rice crop [1]. An annual phytosanitary inspection of rice crops in the Krasnodar Region revealed significant crop losses as a result of blast, from 15 to 45%. The most severe manifestation of the disease is noted during the heading stage period. The development of blast is also influenced by an increased dose of nitrogen fertilizers, which increases the susceptibility of plants to this disease.

Additionally, weeds compete with rice crops for light, mineral nutrition, and space. The chemical protection of crops from diseases and weeds may not always be effective, and there are ecological zones where their chemical treatment is prohibited (territories near the coast). An alternative to chemical plant protection products to combat diseases is breeding varieties with increased resistance. It is possible to enhance plant immunity to pathogens by introducing effective genes [2,3,4,5,6].

It is also possible to reduce the use of herbicides for weed control in rice agrophytocenoses with prolonged flooding (12–14 days) with a deep layer of water. In such conditions, weeds die because they are unable to germinate under it [7,8,9]. Asian scientists have discovered the *Submergence 1 (Sub1)* locus, which controls the trait of submergence tolerance in plants [10,11,12,13,14,15,16]. Xu and co-authors, having studied the genetic mechanisms of tolerance to prolonged flooding in rice with the use of QTL analysis, established that the quantitative trait of this abiotic stressor was controlled by three genes—*Sub1A*, *Sub1B*, and *Sub1C*—located at the Sub1 locus on chromosome 9, near its centromere. The total contribution of these genes to the phenotypic variation of this trait is 70%. Despite the fact that all three genes belong to the B-2 subclass of *ERF* proteins, only the *Sub1A* gene enhances the submergence tolerance of plants [17]. It is known from the literature that this locus is absent in varieties of the Japanese subspecies, which are cultivated in the Russian Federation and other countries [7,8]. Therefore, the question of introgression of this locus into Russian rice varieties arises.

In addition, the strategy of pyramiding both race-specific resistance genes and genes with a wide range of resistance to blast in one genotype is also relevant. This strategy involves the fact that all blast resistance genes (called *Pi*) encode *NBS-LRR* proteins, which consist of a leucine-rich domain (LRRs) and a nucleotide-binding domain (NBS). They perceive pathogen effectors that directly or indirectly lead to ETI (resulting in effector-triggered immunity) [11]. ETI provides strong resistance, including hypersensitivity reactions. However, resistance is limited to several pathogen races and is not long-lasting, since pathogen effectors evolve rapidly [17,18]. In this regard, there is a need to pyramid genes that provide resistance to different races of the pathogen and broad-spectrum genes, which can contribute to long-term disease resistance. In addition, the pyramiding of *Pi* genes and *Sub1A* tolerance genes into domestic rice varieties is promising for the greening of the rice industry. The stimulation of natural protective processes in rice, combining in one genotype genes resistant to the most common and dangerous disease worldwide—blast—as well as genes tolerant to prolonged flooding as a factor in weed control, will reduce the level of pesticide exposure, ensure the restoration of reproductive capabilities of ecosystems, and increase resistance in the cultivation regions of the Russian Federation. The use of DNA markers associated with these traits ensures precise control of the inheritance of target loci, which ultimately reduces the time and material costs while modeling and developing promising genetic plasma. This strategy is promising and aligns with global breeding trends.

The breeding of rice varieties with increased resistance to blast and submergence tolerance will contribute to combating the pathogen *P. oryzae* and segetal vegetation without the use of fungicides and herbicides and will ensure the transition to highly productive and environmentally friendly agro- and aquatic farming, i.e., to a priority area of the Strategy of Scientific and Technological Development of the Russian Federation.

**Purpose of the study:** To breed varieties and breeding material of rice (*O. sativa*) with increased resistance to blast and submergence tolerance as a weed control factor, based on molecular marking methods.

The scientific novelty of the research lies in the fact that, on the basis of molecular marker systems associated with the traits of resistance to blast and submergence tolerance as a factor in weed control, a new breeding material with effective *Pi* genes and the *Sub1A* gene is created by selecting rice plants with donor alleles for the south of Russia. These breeding forms have in their genotype not only target genes that ensure the self-protection of rice plants from the pathogen and submergence tolerance, but also positive biological features (the growing season is 117–120 days, which corresponds to their cultivation in southern Russia), plant height (no higher than 100 cm, which contributes to resistance to lodging), etc. In addition, they have economically valuable features (increased productivity, resistance to seed casting, and resistance to blast), which are important for agricultural producers, and, most importantly, they have improved grain quality, which is important for consumers.

The use of DNA technologies becomes extremely relevant and can serve as an effective auxiliary tool for monitoring and accelerating the breeding process in this area. Their use greatly simplifies the creation of varieties with specified characteristics, as it allows for the selection of elite forms with genes of interest at an early stage of plant development while reducing the volume of breeding nurseries, reducing labor costs, and facilitating the work of the breeder.

## 2. Materials and Methods

Donors for the introduction of blast-resistant genes into the germplasm of Russian varieties such as Flagman, Snezhinka, Novator, Boyarin, Comandor, Kontakt, and the rice lines VNIIR5242, KP-163, KP-24-15, and SP-28/5 were varieties and lines of foreign origin, such as IR-36 (*Pi-ta* gene donor), BL-1 (*Pi-b* gene donor), C101A-51 (*Pi-2* gene donor), and C101LAC (*Pi-1* + *Pi-33* gene donor). The donor of the *Sub1A* gene was the Asian rice variety Khan Dan.

In the applied breeding schemes, plants of donor and recipient forms, as well as hybrid plants of BC-generations, were planted in growing vessels in artificial climate chambers (or in a growing plot, depending on the season of the year) in triplicate with an interval of 3–10 days to synchronize flowering [12]. Hybridization of rice plants was carried out by emasculation and pollination with the “TVELL” method [19]. DNA from the analyzed hybrid samples was isolated from the freshly cut part of the leaf blade of hybrid plants at the stage of 4–5 leaves by the CTAB method [20].

Classical PCR was performed with 40–50 ng of DNA in a final volume of 25 μL. The following composition of the reaction mixture was used: 0.05 mM deoxyribonucleoside phosphates (dNTPs), 0.3 μM of each primer, 25 mM KCL, 60 mM Tris-HCL (pH 8.5), 0.1% Triton X-100, 10 mM 2-mercaptoethanol, 1.5 mM MgCL_2_, and 1 unit of Taq-polymerase.

DNA amplification was carried out under the following conditions: initial DNA denaturation at 94 °C—4 min, followed by 30 cycles of 1 min—denaturation at 94 °C, 1 min—primer annealing at 55 °C, 1 min—elongation at 72 °C, and a last synthesis cycle of 5 min at 72 °C.

Real-time PCR was carried out in a PCR mixture final volume of 25 μL, which contained PCR buffer, 1.25 units of Taq-polymerase, 100 μmol dNTP, 10 pmol of each primer, 5 pmol of each DNA probe, and 5 μL of DNA solution. The primers used in the assay are represented in Table 1.

The amplification reaction products were separated by electrophoresis in an 8% polyacrylamide gel (PAAG) and a 2% agarose gel. Visualization of the result of electrophoretic separation of PCR products was performed using ethidium bromide (BrEt, 2,7-diamino-10-ethyl-9-phenylphenatridinium bromide, homidium bromide) [21].

Identification of *Pi-ta* and *Pi-b* genes was conducted with the use of intragenic molecular markers. PCR real-time testing was performed using technology developed by the Laboratory of Information, Digital, and Biotechnologies of the FSBSI “Federal Scientific Rice Centre” and the Laboratory of Genome Analysis of the FSBSI “All-Russian Research Scientific Institution of Agricultural Biotechnology” jointly. The amplification was carried out in the thermocycler QuantGene 9600 (Bioer, Hangzhou China) under the following conditions: initial DNA denaturation at 95 °C—10 min, followed by 40 cycles of 10 s—denaturation at 94 °C, 30 s—primer annealing at 60 °C, and 5 s—elongation at 72 °C [22].

For precise detection of the *Pi-ta* gene, the following primers and DNA probes were synthesized:

– PaF (5′-CCA GGT TAC AAC TTA CAA GGA-3′);
– PaR (5′-AGA GGA TTC CGG TAG CAT ACA-3′);
– PaG (5′-FAM-CTT CTA TCT TTA CCT GCT ATG CAT-RTQ1-3′);
– PaT (5′-R6G-CTT CTA TCT TTA CCT TCT ATG CAT C –BHQ2-3′) (Syntol, Moscow, Russia).

For identification of the *Pi-b* gene allelic state, the following primers and DNA probes were synthesized:

– PbF1(5′-GAA CAG CTT GCT CGG AAT CCA A-3′);
– PbR2 (5′-TAC TGC ATT GTG CAG CTT GTG C-3′);
– PbR3 (5′-TAC ATC GAC CAG CTA TTT GCC G-3′);
– PbR (5′-R6G-TGC CGG ACC TGA GCT GCC ACA TAT GC-BHQ1-3′);
– PiBD2 (5′-ROX-GCC GTG CTC CAT ACT ATC CTA CAA GTG A-BHQ2-3′) (Syntol, Moscow, Russia).

While conducting the amplification of the *Sub1A* gene, a microsatellite marker with specific primers RM7481 taken from the NCBI database, associated with the locus responsible for submergence tolerance, was used as follows:

– RM7481F (5′-CGACCCAATATCTTTCTGCC-3′);
– RM7481R (5′-CATTGGTCGTGCTCAACAAG-3′).

DNA amplification of the *Sub1A* gene was performed using the following protocol: initial DNA denaturation at 94 °C—5 min, following by 35 cycles of 35 s—denaturation at 94 °C, 45 s—primer annealing at 60 °C, 30 s—elongation at 72 °C, and the last synthesis cycle iof 5 min at 72 °C. PCR was conducted in the thermocycler GeneExplorer GE-96G (Bioer, China). The amplification reaction products were separated by electrophoresis in a 2% agarose gel [21].

For biometric analysis, 15 typical plants of promising samples were selected, distinguished by their economically valuable characteristics and resistance to blast, in four repetitions.

Statistical processing of the obtained data was carried out using Microsoft Office Excel 2010 and STATISTICA 10.0 for Windows application software packages. To assess the economic and valuable characteristics and resistance to blast, the studied rice breeding samples with *Pi* genes were sown on a rice irrigation system according to the predecessor of perennial grasses in the A.I. Maistrenko Federal State Budgetary Educational Institution of the Russian Academy of Sciences “Krasnoarmeysky” branch of the FSBSI “FSC of Rice” of the Krasnoarmeysky district and in the separate division “Proletarskoe” of the Agricultural Research Centre “Donskoy” of the Rostov region. After cultivating perennial grasses, nitrogen accumulates in the soil. To improve plant growth and development and increase yield and product quality, agricultural producers additionally carry out nitrogen fertilization during the tillering phase (N46 kg/ha according to the active substance). Under such excessive nitrogen content, combined with favorable weather conditions (temperature 26–28 °C and air humidity of at least 90–95%), the causative agent of blast can develop at lightning speed and cause the mass death of rice plants. When assessing resistance in the field, breeding samples with a disease development index (IDD) of no more than 25% are selected. The remaining samples are rejected.

When studying rice samples in a growing area in metal vessels, an assessment of resistance is carried out using artificial contamination.

The assessment of the degree of damage to plants (in percentages) was conducted on the 14th day after inoculation, in accordance with the express method for assessing rice varietal resistance to blast. The assessment was performed taking into account two indicators: the type of reaction (in points) and the degree of damage (in percentages), using the following ten-point scale of the International Rice Research Institute [23]:

– Resistant: 0–1 points—no damage, small brown spots, covering less than 25% of the total leaf surface;
– Medium-resistant: 2–5 points—typical elliptical blast spots, 1–2 cm long, covering 26–50% of the total leaf surface;
– Susceptible: 6–10 points—typical blast spots of elliptical shape, 1–2 cm long, covering 51% or more of the total leaf surface.

The intensity of disease development (IDD, %) was calculated by the following formula:

IDD = ∑ (a × b)/ n × 9

where IDD—intensity of disease development, %; ∑ (a × b)—the sum of the products of the number of infected plants multiplied by the corresponding damage point; and *n* is the number of recorded plants, pcs.

Depending on the damage point, all varieties are conventionally divided into 4 groups: resistant, intermediate, susceptible, and strongly susceptible.

## 3. Results and Discussion

Due to the fact that blast (the causative agent is *P. oryzae*) is considered to be one of the most harmful rice diseases worldwide, accelerating some stages while developing resistant genotypes using modern molecular marker methods, along with increasing yields, is highly relevant.

For the successful implementation of programs to create pathogen-resistant rice varieties, the breeder encounters the primary task: to select effective resistance genes for a specific region and introgress them into highly productive domestic germplasm. Therefore, at the first stage, we conducted such studies and identified effective genes for the south of Russia [17,18,19,20]. The *Pi-ta* and *Pi-b* genes have been sequenced. The *Pi-1* and *Pi-2* genes belong to the genes that confer race-specific resistance to the Krasnodar population of the pathogen [1,2,3,4,5,6,22].

In this regard, based on the use of DNA marker breeding technology and hybridization methods, we have been implementing a program since 2007 to introduce and combine the blast resistance genes *Pi-1*, *Pi-2*, *Pi-33*, *Pi-ta*, and *Pi-b* into highly productive domestic rice varieties adapted to agro-climatic conditions of rice growing in the south of Russia.

**Figure 1:**
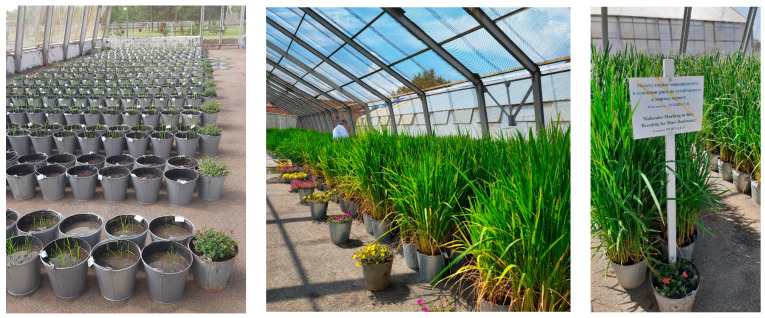
Reproduction of hybrid and backcross self-pollinated rice lines with Pi and Sub1A genes.

Every year, up to 3000 hybrid plants with *Pi* genes obtained from crossing rice lines KP-163 × A-51 (*Pi-2* gene donor), KP-24-15 × C101 Lac (*Pi-1 + Pi-33* genes donor), VNIIR5242 × C101 Lac (*Pi-1 + Pi-33* genes donor), KP-24-15 × C101 Lac (*Pi-1 + Pi-33* genes donor), and KP-24-15 × Bl-1 (*Pi-b* gene donor) were propagated on the growing plot of the Federal Scientific Rice Centre [(Flagman × Virgo/Flagman)] × [(Flagman × A-51/Flagman)], [(Snezhinka × Virgo/Snezhinka)] × [(Snezhinka × Bl-1/Snezhinka)], and Flagman × Virgo, SP 28-5/KP-30 × Virgo/KP-30. In addition, according to the program of pyramiding genes for resistance to blast and submergence tolerance, up to 600 hybrid plants of F_2_ and subsequent generations with the *Pi* and *Sub1A* genes in the genotype have been propagated (Figure 1).

**Figure 2:**
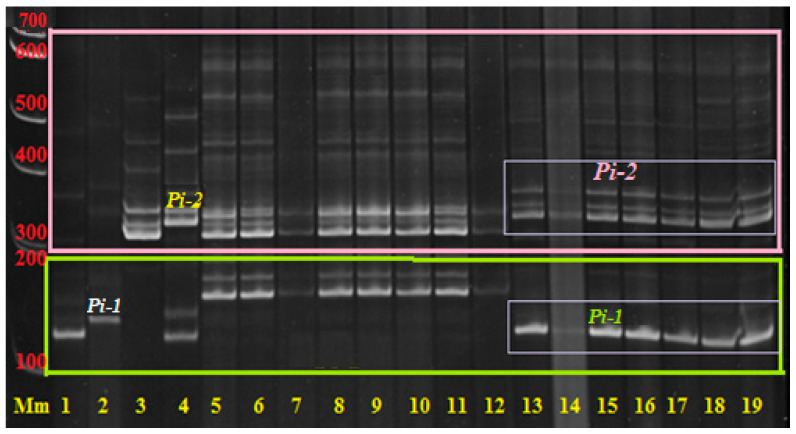
Multiplex PCR of genomic DNA of hybrid F_4_ rice plants by (RM224, RM144) + (SSR140, RM 527) loci in an 8% polyacrylamide gel (PAAG). Note: Mm—molecular mass marker 100 bp + 1.5 Kb + 3 Kb (SybEnzyme); 1—maternal form analyzed for presence of *Pi-1* gene (with recessive allele “aa” of *Pi-1* target gene—124 bp; 2—rice line C101-LAC (donor of *Pi-1* gene with donor allele “AA”—158 bp); 3—maternal form analyzed for *Pi-2* gene presence (with recessive allele “aa” of *Pi-2* target gene—313 bp; 4—line C101LAC-A-51 (donor of *Pi-2* gene with donor allele “AA”—334 bp); 5…19—electrophoretic lanes with PCR products of analyzed F_4_ rice plants of hybrid combination [Flagman/C101Lac × Flagman/Virgo] × [Flagman/A-51 × Khan Dan]: 188-1, 188-2, 188-3, 188-4, 188-5, 188-6, 188-7, 188-8, 189-2, 189-3, 189-5, 189-6, 189-7, 189-8, and 189-10.

The DNA of each plant is analyzed to identify the genes of interest in their genotypes using molecular SSR markers (Figure 2 and Figure 5). For further work, plants with target genes in the genotype and positive morphometric characteristics are selected for field trials to assess economically valuable traits and resistance to blast.

The effectiveness and acceleration of the breeding scheme are provided by marker control of these genes. Figure 2 shows the results of some PCR analyses in an 8% PAAG for the identification of target blast resistance genes *Pi-1* and *Pi-2* in hybrid material.

Figure 2 shows the results of PCR diagnostics of F_4_ rice plants of the hybrid combination [Flagman/C101Lac × Flagman/Virgo] × [Flagman/A-51 × Khan Dan] for the presence of *Pi-2* and *Pi-1* genes. It can be seen from the gel picture that the analyzed plants No. 13…19 have in their genetic profile PCR products with a size of 334 bp, which corresponds to the donor allele of the *Pi-2* gene, and 158 bp, which also corresponds to the donor allele of the *Pi-1* gene, i.e., they are homozygous for these genes and provide resistance to blast. Sample Nos. 5…12 have recessive alleles (313 and 124 bp, respectively) of both genes, i.e., they are susceptible to the disease and excluded from subsequent work.

**Figure 3:**
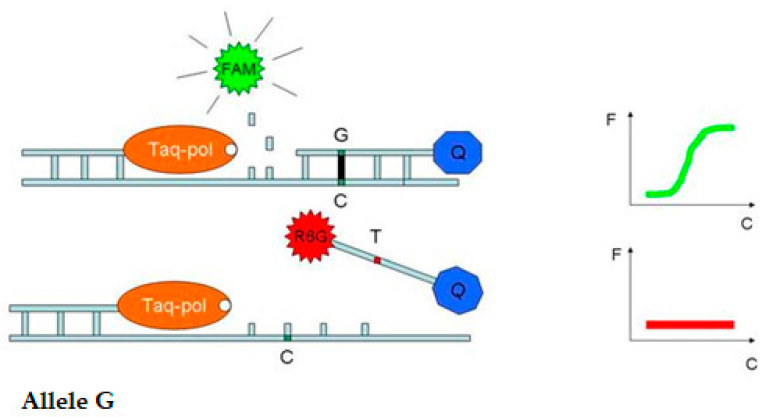
The scheme of allele-specific real-time PCR for detection of polymorphism G/T in the *Pi-ta* gene (the example of detection of dominant homozygote) (photo [22]). Note: FAM—the dye of DNA probe for resistant allele: R6G—the dye of DNA probe for susceptible allele; Q—quencher of fluorescence; Taq-pol—Taq polymerase; green fluorescence growth plot demonstrate the presense of resistant allele in FAM detection channel; red luorescence growth plot demonstrate the presense of susceptinle allele in R6G detection channel; Allele G—dominant allele of the *Pi-ta* and *Pi-b* genes.

For the detection of *Pi-ta* and *Pi-b* genes in experimental rice plants, we used the real-time PCR method. The technology of this method was developed by the staff of the laboratory of genome analysis of the “All-Russian Research Institute of Agricultural Biotechnology” in collaboration with our laboratory in order to transform the available marker systems for these genes into real-time PCR format with TaqMan probes, which allow for the identification of various allelic states of the *Pi-ta* and *Pi-b* genes. The essence of the method is that during annealing, the probe is hybridized with the target DNA sequence according to the principle of complementarity. During elongation, Taq DNA polymerase cleaves the hybridized probe; as a result, the fluorophore and the quencher are spatially separated, which leads to an increase in fluorescence in each PCR cycle (in blast-resistant forms). A probe containing a different variant of polymorphism (in susceptible forms) inefficiently hybridizes with the target sequence at a determined temperature; therefore, it remains undisturbed, and no fluorescence growth is observed (Figure 3). In heterozygotes, both probes participated in the reaction, and an increase in fluorescence was detected in two detection channels.

The polymorphism of the *Pi-b* gene was determined according to a similar scheme using real-time PCR. Primers and probes were added to the reaction mixture, some of which are specific to the sequence of the dominant resistance gene, while others are specific to a different sequence of susceptible forms. The formation of the product in PCR indicates the presence of a particular sequence and was recorded as an increase in fluorescence, in one of the channels in the case of homozygotes and in two channels in the case of heterozygotes [22].

To conduct studies using PCR analysis with electrophoretic detection, control forms of rice (IR-58 and Moroberekan) containing the dominant *Pi-ta* and *Pi-b* genes, respectively, and the Boyarin variety, with recessive alleles of these genes, were used. The nucleotide sequence of the obtained PCR products was determined using an ABI PRISM 3130XL genetic analyzer (the Centre of Collective use of “Biotechnology”, “All-Russian Research Institute of Agricultural Biotechnology”).

**Figure 4:**
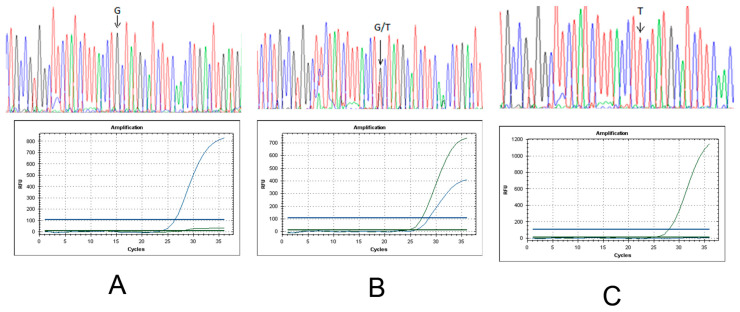
Chromatograms of DNA-sample sequences with designation of a polymorphism point of the *Pi-ta* gene and corresponding fluorescence growth plots: (**A**)—in FAM-channel for homozygote G, (**B**)—in FAM and R6G channels for heterozygotes G/T, and (**C**)—in R6G channel for homozygote T (photo [22]).

When testing the developed technology on control samples of IR-58 or IR-36 containing the dominant *Pi-ta* gene and the Boyarin variety with a recessive allele of this gene, DNA analysis based on the use of real-time PCR showed an increase in fluorescence in the IR-58 (G-state) only in the FAM channel, and in the Boyarin (T-state), an increase in fluorescence was observed only in the R6G channel. Hybrid heterozygotes with both alleles showed growth in two fluorescence channels, FAM and R6G (Figure 4). This confirms the allele-specific hybridization of the probes and the correct operation of the method on control DNA samples.

The analysis of control samples for the *Pi-b* gene presence, where the Moroberekan or BL-1 rice variety was used as the dominant gene donor and the Boyarin variety was also used as the recessive allele donor, showed that in the Moroberekan variety, fluorescence growth was observed only in the ROX channel. In susceptible homozygous plants, fluorescence growth was present only in the R6G channel. Heterozygotes showed fluorescence growth in two channels, ROX and R6G.

After obtaining these results, we involved this technology in the breeding process to identify the *Pi-ta* and *Pi-b* genes. Its use makes it possible to quickly (in a short time) analyze large volumes of experimental breeding forms for the presence of target genes in the genotype, significantly speeding up and facilitating large-scale genetic analyses, ensuring the reliable obtaining of unique characteristics of each genotype. In 1.5 h of working time, 94 experimental samples can be diagnosed using this technology. Classical PCR, which takes a long time, can analyze no more than 17 samples in one analysis (PAAG plate). In addition, a laborious electrophoresis method using toxic carcinogens, such as ethidium bromide (BrEt), is required to detect PCR products. In this regard, we identified introgressed and pyramided *Pi-ta* and *Pi-b* genes in the studied experimental rice plants based on real-time PCR.

**Figure 5:**
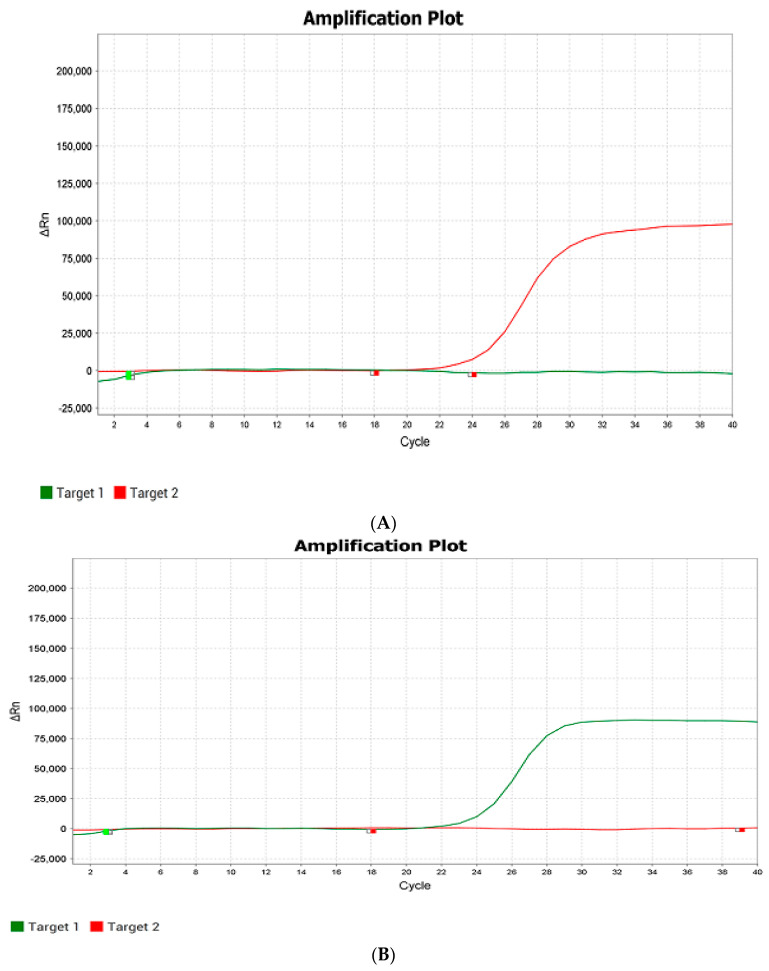

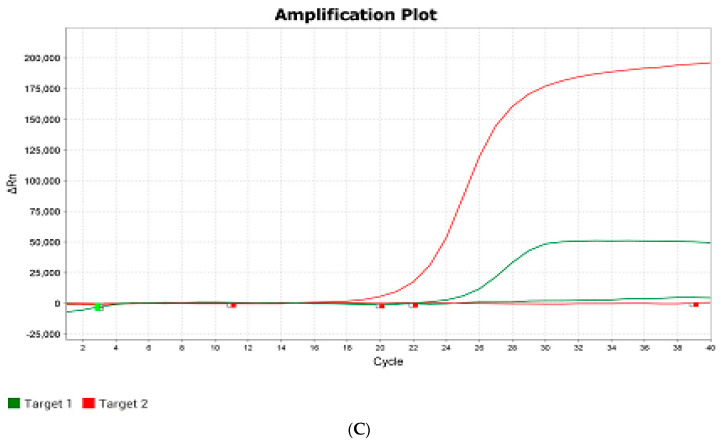
Fluorescence growth plot in real-time PCR as a result of *Pi-b* gene detection: (**A**)—hybrid rice plant by VIC channel (recessive homozygote with recessive allele (red color)), (**B**)—hybrid rice plant by ROX channel (dominant homozygote with donor allele (green color)), and (**C**)—hybrid rice plant by ROX and VIC channels (heterozygote). The photo is taken from the PCR program. Note: 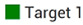
—VIC (detection channel/green color)—recessive allele; 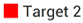
—ROX (detection channel/red color)—donor allele. The quantity of amplification cycles is plotted by the *X*-axis, and ΔRn (the increment of fluorescence signal at every moment) is plotted by the *Y*-axis.

An example of real-time PCR analysis is shown in Figure 5.

The color of the detection channel is selected automatically in the program on the QuantGene 9600 (Bioer, Hangzhou China) device. From the analyzed hybrid plants obtained at the growing plot of the Federal Scientific Rice Centre in 2024, 17 homozygotes with the dominant allele of the *Pi-b* gene were identified; 254 plants were heterozygous, and 3 plants were recessive homozygotes, according to the results of real-time PCR. Based on the results of automatic detection of the analyzed hybrid rice samples for the presence of the *Pi-ta* gene in the genotype, 254 homozygotes with a recessive allele of the *Pi-ta* gene and 3 heterozygotes were identified.

**Figure 6:**
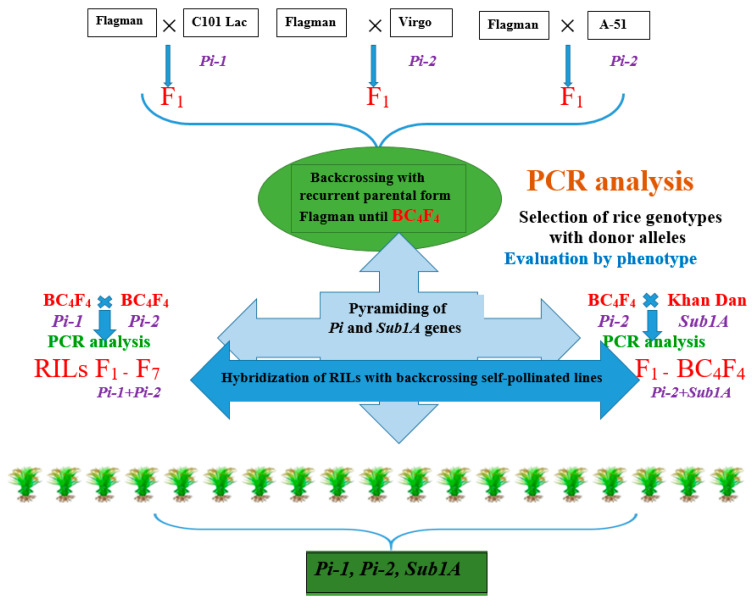
Scheme of introducing the submergence tolerance Sub1A gene into domestic rice varieties using MAS.

At the same time, in 2013, the hybridization of domestic rice variety Flagman with donors of blast resistance genes *Pi-1* and *Pi-2* [Flagman × C101Lac], [Flagman × Virgo], and [Flagman × A-51] was conducted (Figure 6). The first generation in 2013 was characterized by a high degree of sterility (90–95%). In the second generation in 2014, a broad spectrum of splitting was observed in terms of the growing season, plant height, panicle length and shape, number of spikelets, and spinousness. Four backcrosses for each hybrid combination and self-pollination were performed to transform the donor alleles of transferred genes into the homozygous state. Then, the pyramiding of the *Pi-1* and *Pi-2* genes was conducted, as a result of which RILs were obtained, and the pyramiding of the *Sub1A* gene with *Pi-2* genes was performed, as a result of which the BC_4_F_4_ populations were obtained. In order to pyramid several blast resistance genes, RILs were crossed with BC_4_F_4_ populations. The obtained F_1_ generation was involved in a series of backcrosses and self-pollination. So the BC_4_F_4_ population with *Pi-1*, *Pi-2*, and *Sub1A* genes was obtained at this point.

In the BC_4_F_4_ generation in 2021, a broad spectrum of splitting was observed in terms of the vegetation period, plant height, panicle length and shape, number of spikelets, and spinousness (Table 2).

**Figure 7:**
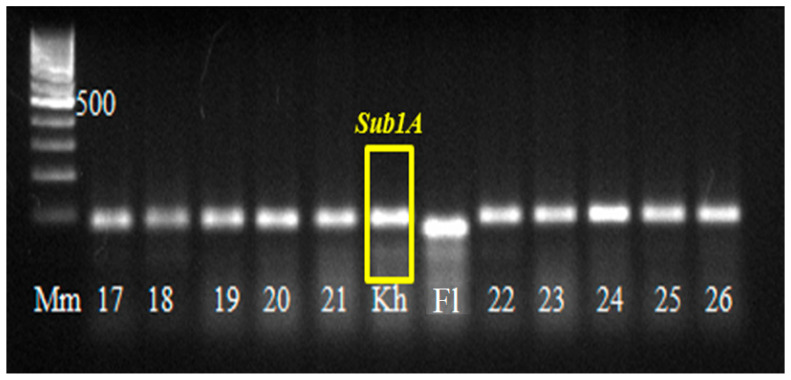
Results of PCR analysis of hybrid BC_4_F_4_ rice plants of hybrid combination [Flagman/C101Lac × Flagman/Virgo] × [Flagman/A-51 × Khan Dan] with the use of SSR marker RM7481. Note: Mm—molecular mass marker 100 bp + 1.5 Kb + 3 Kb (SybEnZyme, Moscow, Russia); 17…26—electrophoretic lanes with the analyzed hybrid rice plants of the combination [Fl/C101Lac × Fl/Virgo] × [Fl/A-51 × Khan Dan]; Kh is the electrophoretic lane of the Khan Dan variety, the donor of the *Sub1A* gene; and Fl is the electrophoretic lane of the Flagman rice variety.

**Figure 8:**
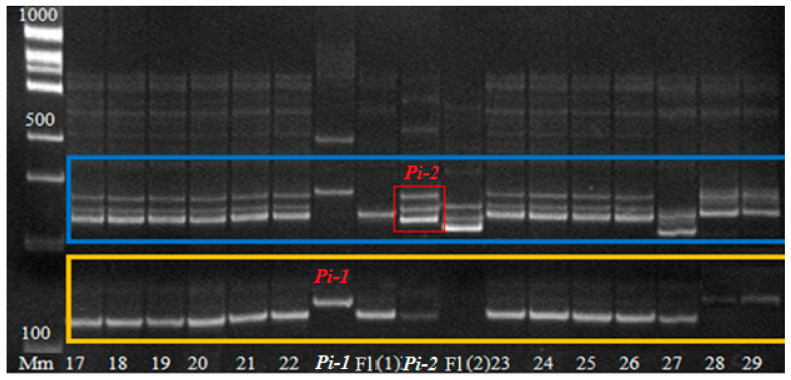
Detection of PCR products BC_4_F_4_ rice plants of hybrid combination [Flagman/C101Lac × Flagman/Virgo] × [Flagman/A-51 × Khan Dan] for the presence of *Pi-1*, *Pi-2* genes. Note: Mm—molecular mass marker 100 bp + 1.5 Kb + 3 Kb (SybEnzyme), 17…29—electrophoretic lanes with PCR products of analyzed rice plants of hybrid combination [Flagman/C101Lac × Flagman/Virgo] × [Flagman/A-51 × Khan Dan], *Pi-1*—rice line C101-LAC (donor of *Pi-1* gene), Fl (1)—rice variety Flagman analyzed for the presence of *Pi-1* gene, Fl(2)—rice variety Flagman analyzed for the presence of *Pi-2* gene, *Pi-2*—line C101LAC-A-51 (donor of *Pi-2* gene).

In 2021, a BC_4_F_4_ population with pyramided genes *Pi* and *Sub1A* was obtained, which was tested by PCR analysis for the presence of *Sub1A*, *Pi-1*, and *Pi-2* genes in the genotype (Figure 7 and Figure 8). Of 180 hybrid plants, 43 plants with three target genes were isolated and tested by a laboratory express method for submergence tolerance and resistance to blast. The phenotype assessment fully confirmed the data from the molecular genetic analysis. These samples were studied in the breeding nursery for economically valuable traits. The best samples are represented in Table 2.

Figure 7 shows that all hybrid plants No.17…26 have the dominant *Sub1A* allele in their genotype.

Figure 8 shows the presence of the *Pi-1* and *Pi-2* genes in the analyzed samples. As a result of the analysis shown in Figure 7 and Figure 8, it can be concluded that hybrid plants under No. 17…22 have the *Pi-2* blast resistance gene and the *Sub1A* submergence tolerance gene in the genotype [23,24,25]. Then they will be tested in field trials for economically valuable traits. The best samples will be transferred to the State Variety Testing system.

The creation and implementation of rice genotypes with genes for resistance to blast (*Pi*), as well as tolerance to prolonged flooding (*Sub 1A*), will reduce the level of pesticide exposure to agricultural land as well as crop losses due to rice plants affected by blast and in competition with weeds in the regions of cultivation in Russia. Only such a breeding strategy will ensure the transition to highly productive and environmentally friendly agro and aquatic farming, a priority area of the Strategy of Scientific and Technological Development of the Russian Federation.

**Figure 9:**
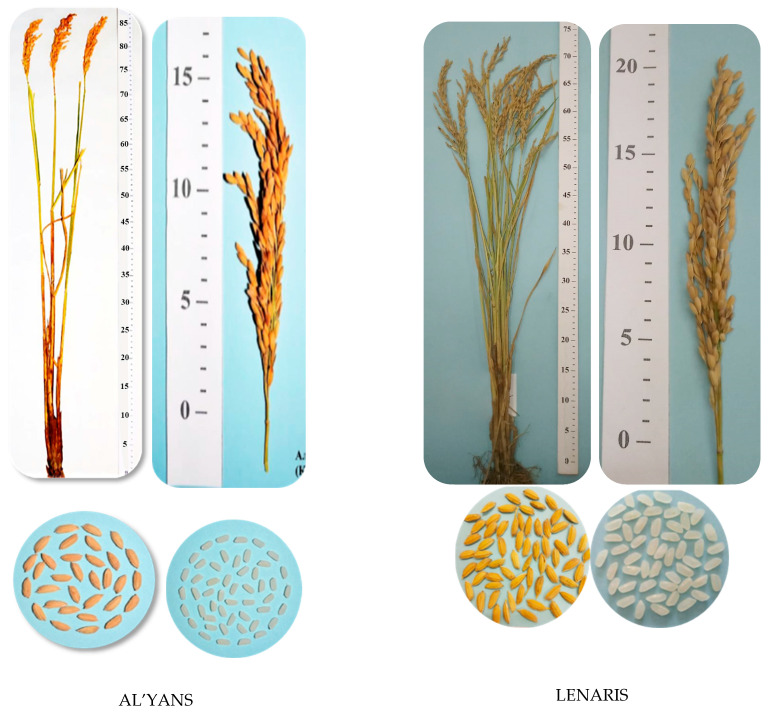
Rice varieties Al’yans and Lenaris, breed by DNA marking of target genes technology.

To diversify the rice gene pool with the introgressed and pyramided blast resistance genes *Pi-1*, *Pi-2*, *Pi-33*, *Pi-ta*, *Pi-b*, extensive breeding material was obtained, which, according to the schemes of the breeding process, had been studied for economically valuable traits and field resistance to blast on the rice irrigation system of the FSI Rice Breeding Farming “Krasnoarmeyskiy”, named after A.I. Maistrenko and based on the predecessor of perennial herbs. As a result of severe rejection of plant material during the breeding process, the KP-17114 variety was released in 2014, and the KP-30 variety was released in 2015, both of which were transferred to the State Variety Testing system and called Al’yans and Lenaris, respectively (Figure 9).

**Figure 10:**
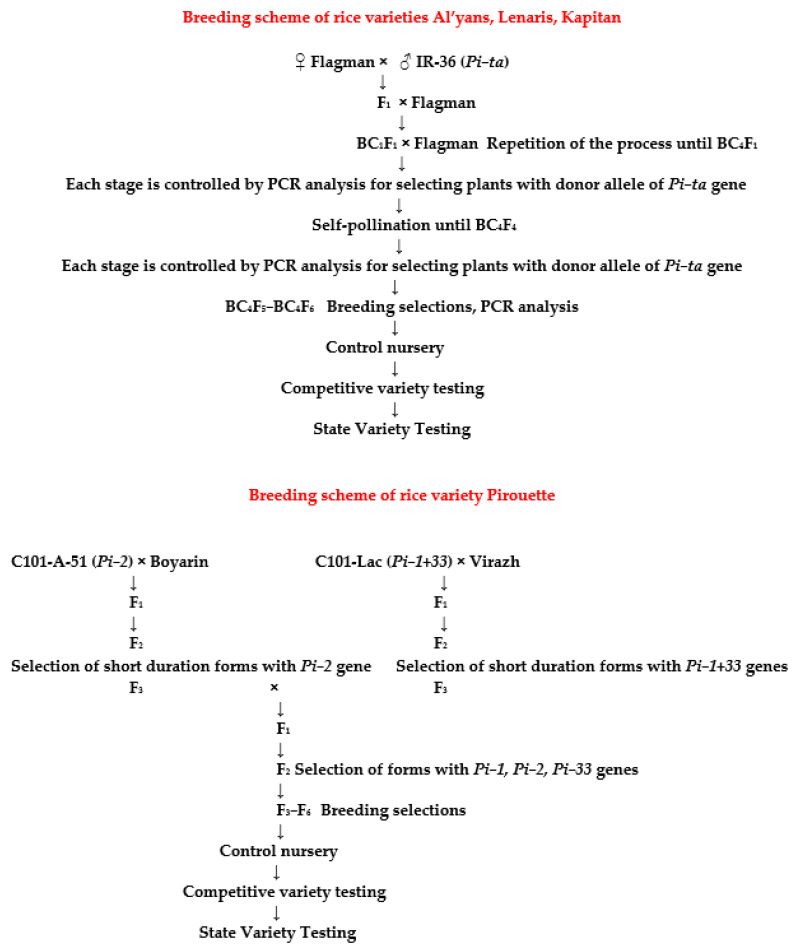
Breeding scheme of rice varieties Al’yans, Lenaris, Kapitan, and Pirouette.

The breeding scheme of rice varieties Al’yans, Lenaris, Kapitan, and Pirouette is represented in Figure 10.

After crossing the maternal form (domestic rice variety Flagman) with the donor of the *Pi-ta* gene (rice variety IR-36), the F_1_ generation was obtained, which was used in backcrosses with the recipient parental forms (domestic rice varieties). It should be noted that F_1_ plants had a high degree of sterility (up to 95%). After the first series of backcrosses in 2008 in artificial climate chambers, the BC_1_ and BC_2_ generations were obtained. In BC_1_ populations, fertility increased and averaged about 50%. Starting from the first backcross, marker control was conducted for the presence of transferable donor alleles in the hybrid offspring.

In 2009, plants of the BC_3_ and BC_4_ generations were obtained. Among these plants were forms with the smallest vegetative period and the highest fertility of the panicle. From the BC_4_F_1_ stage (the first self-pollination of rice plants, which makes it possible to transfer the donor allele to a homozygous state), individual selection was performed. Plants were selected that were closest in morphotype to the recipient parent form and, in addition, bore donor genes of resistance to the pathogen in a homozygous state [5].

For each combination, four backcrosses were carried out, since it is known that the restoration of the genome of the recurrent parent (RP) in backcrosses in BC_4_ is 96.9% [1].

This strategy was used not only to increase the immunity of rice varieties already in production to the disease, but also to obtain a wide variety of elite genotypes with a new morphotype with increased yields, quality, and resistance to blast, as well as to facilitate their accelerated introduction into production.

Selected BC_4_F_4_ plants with positive morphometric characteristics were transferred to a breeding nursery to study their economically valuable traits. Then, BC_4_F_5_-BC_4_F_6_ populations were obtained, which were evaluated for resistance to blast and lodging under field conditions. Also, while estimating a vegetation period of no more than 125 days, plant heights of no more than 105 cm were taken into account. The best rice forms were transferred to the control nursery, where three rice samples from the hybrid combination (Flagman × IR-36): KP-17114 (Al’yans), KP-30 (Lenaris), and KP-23 (Kapitan) were selected. As a result of trials, these samples showed high yield and resistance to blast and lodging, and were transferred to the State Variety Testing system, where they successfully passed estimation and then were zoned. The characteristics of these varieties are shown below.

The rice variety Al’yans with the *Pi-ta* blast resistance gene was obtained from the hybrid population Flagman/IR-36 (*Pi-ta*) × Flagman. The species is *O. sativa*, the subspecies is subsp. *japonica*, and the botanical variety is var. *italica* Alef. It belongs to the medium-ripened group; the growing season from the bay to full ripeness is 115–117 days. The height of the plants is 100–105 cm. The length of the panicle is 17–19 cm. The mass of grain from the panicle is 3.5–4.0 g. The mass of 1000 grains is 29.0 g. The total outcome of cereals is 92%. The whole kernel content in cereals is 86.4–89.2%, and the productivity is 9.1 t/ha, which exceeds the Flagman standard variety (7.2 t/ha) by 1.9 t/ha. The resistance to lodging is 7 points out of 10, and the resistance to blast, as measured by the index of disease development, is 14.7–17.8 %.

The Lenaris rice variety with the *Pi-ta* gene was selected from the hybrid population Flagman/IR-36 (*Pi-ta*) × Flagman. The species is *O. sativa*, the subspecies is subsp. *japonica*, and the botanical variety is var. *italica* Alef. It belongs to the middle-aged group. The variety belongs to the medium-ripened group; the growing season from bay to full ripeness is 115–117 days. The height of the plants is 85–90 cm. The length of the panicle is 17–20 cm, compact and slightly inclined. The weight of the grain from the panicle is 3.7–4.2 g. The weight of 1000 grains is 30.2–30.4 g. The vitreous content is 85–90%, filminess—19.8%, and fracturing—19%. The total outcome of cereals is 71.2–72.2%. The content of the whole kernel in cereals is 86.4–89.2%. The amylose content is 20.2%. The cereal is white. The yield is 9.4 t/ha, which exceeds the Flagman standard variety (7.2 t/ha) by 2.2 t/ha. It is resistant to lodging (8 points out of 10) and resistant to blast (index of disease development, 9.9–17.8%).

Earlier, a medium-ripened Pirouette rice variety carrying blast resistance genes was breed in the Agricultural Research Centre “Donskoy” in collaboration with the Federal Scientific Rice Centre. It was obtained by stepwise hybridization and marker selection from the hybrid population (C101-A-51 (*Pi-2*) × Boyarin) × (C101-Lac (*Pi-1 + 33*) × Virazh). The variety was entered into the Register of Breeding Achievements of the Russian Federation in 2021. The variety belongs to the medium-ripened group, and the growing season from bay to full ripeness is 124 days. The plants are medium-sized, with an average height of 88 cm, and have a compact shrub form with vertical arrangement of leaves and panicles. The panicle is erect, compact, 17.5 cm long, and bears 162 spikelets. The average weight of 1000 grains is 31.6 g. The grain is white and vitreous (94.3%). The filminess of the grain is 21.3%, the grain yield is 72.2%, and the whole kernel is 78.7%. On average, over the years of competitive testing, the yield of the Pirouette variety was 9.57 t/ha, which is 1.13 t/ha higher than that of the Yuzhanin standard variety. It is resistant to blast (index of disease development %). The maximum productivity is 10.05 t/ha. The variety is resistant to blast, lodging, and shedding, as well as being cold-resistant. At the same time, production costs are significantly reduced, since it is possible to reduce the consumption rate of fungicides.

The Kapitan variety was breed from the hybrid population Flagman/IR-36 (*Pi-ta*) × Flagman in the Agricultural Research Centre “Donskoy” in collaboration with the Federal Scientific Rice Centre by triple backcrossing and marker breeding from the Flagman × IR-36 hybrid population using PCR analysis. The variety carries the blast resistance gene *Pi-ta*. The variety belongs to the medium-ripened group, with a growing season from bay to full ripeness of 120 days. The plants are medium-sized, with an average height of 90 cm. The panicle is semi-inclined, compact, 17.0 cm long, and bears 140–150 spikelets. The spikelets are elongated, 9.5 mm long and 3.6 mm wide. The average weight of 1000 grains is 35 g. The ratio of grain length to width is 2.6. The grain is white and vitreous (93.3%). The filminess of the grain is 20.5%. The yield of cereals is 71.5%, and the whole kernel is 86.4%. The variety is resistant to blast, lodging, and shedding. On average, over the years of competitive testing, the yield of the Kapitan variety was 8.13 t/ha, which is 0.64 t/ha higher than that of the standard Yuzhanin variety. The maximum realized yield is 9.0 t/ha. It is resistant to blast (index of disease development of 11.6–17.4%).

The higher yield of this variety is due to a more lacerated panicle than that of the standard, as well as an increased grain weight.

These varieties are included in the Register of Breeding Achievements of the Russian Federation and are widely used in production.

## 4. Conclusions

1. As a result of the scientific work carried out, a large volume of the rice gene pool has been created with genes for blast resistance and submergence tolerance genes as a factor in weed control.

2. The created varieties Al’yans, Lenaris, Kapitan, and Pirouette with increased resistance to blast exceed the standards by 0.64–2.2 t/ha, and their involvement in production allows for obtaining additional products by increasing yields in the amount of about RUB 80 thousand per hectare. In addition, their cultivation will allow the avoidance of epiphytotic development of disease, as well as reduced herbicide treatments. It will help to preserve rice yields and provide an opportunity to obtain environmentally friendly agricultural products.

3. This strategy provides rice farms with annual savings of RUB 5000 to 7000 per hectare. When converted to the area of cultivation (in the Krasnodar region, it is 117.4 thousand hectares), this amounts to between RUB 585 and 820 million annually.

## Figures and Tables

**Table 1:** Nucleotide sequence of primers for identification of blast resistance gene.

Marker Name/Genetic Distance	Gene	Nucleotide Sequence of Primers (5′→3′)
Rm 224	(0.6 cM)	F: ATC GAT CGA TCT TCA CGA GG
	*Pi-1*	R: TGC TAT AAA AGG CAT TCG GG
		F: TGCCCTGGCGCAAATTTGATCC
Rm 144	(0.1 cM)	R: GCTAGAGGAGATCAGATGGTAGTGCATG
Rm 527	(1.1 cM)	F: GGC TCG ATC TAG AAA ATC CG
	*Pi-2*	R: TTG CAC AGG TTG CGA TAG AG
		F: AAG GTG TGA AAC AAG CTA GCA A
		R: TTC TAG GGG AGG GGT GTA GAA
SSR140	(3.1 cM)	
Rm 310	(2.3 cM)	F: CCG GCG ATA AAA CAA TGA G
	*Pi 33*	R: GCA TCG GTC CTA ACT AAG GG
RM 72	(3.6 cM)	F: CCG GCG ATA AAA CAA TGA G
		R: GCA TCG GTC CTA ACT AAG GG
Pi-ta	(intragenic)	F1: GCC GTG GCT TCT ATC TTTA CAT G
	*Pi-ta*	R1: ATC CAA GTG TTA GGG CCA ACA TTC
		F2: TTG ACA CTC TCA AAG GAC TGG GAT
		R2: TCA AGT CAG GTT GAA GAT GCA TCG A
Pi-b	(intragenic)	F1: GAA CAG CTT GCT CGG AAT CCA
	*Pi-b*	R2: TAC TGC ATT GTG CAG CTT GTG
		R3: ATA CAT CGA CCA GCT ATT TGC C

**Table 2:** Selected BC_4_F_4_ hybrid plants from crossing submergence-resistant samples with Flagman, 2021.

№, Hybrid	Vegetation Period, Days	Plant Height, Cm	Panicle Length, Cm	Number of Grains in Panicle, Pcs.	Mass of 1000 Grains, G	IDD,%	Submergence Tolerance, +/−
Flagman, standard	112	97.5	16.5	110	26.8	56.8	−
156(F×C101) *	120	108.0	21.5	146	27.0	12.1	−
174 (F×C101)	118	96.7	17.3	145	21.6	11.6	−
230 (F×V)	121	81.2	16.5	109	25.3	4.2	−
390 (F×V)	122	82.0	17.5	122	27.7	5.7	−
73 (F×A-51)	120	95.4	15.1	151	24.4	6.7	−
75 (F×A-51)	119	97.2	19.0	138	29.3	7.1	−
681 (Pi-1×Pi-2)	123	96.5	17.2	159	32.0	5.5	−
393 (Pi-1×Pi-2)	121	97.3	14.9	152	25.2	6.4	−
563 (Pi-2×Sub1A)	120	87.3	16.3	132	31.3	9.7	+
597 (Pi-2×Sub1A)	122	90.4	17.5	134	29.4	11.1	+
904 (Pi-1/Pi-2×Pi-2/Sub1A)	120	93.2	17.8	145	30.2	2.2	+
875 (Pi-1/Pi-2×Pi-2/Sub1A)	123	96.1	19.1	146	30.4	4.6	+

Note *: (F×C101)—Flagman × C101Lac, (F×V)—Flagman × Virgo, (F×A-51)—Flagman × A-51, (*Pi*-*1*×*Pi-2*)—Flagman/C101Lac × Flagman/Virgo], (*Pi-2*×*Sub1A*)—Flagman/A-51 × Khan Dan, (*Pi-1*/*Pi-2*×*Pi-2*/*Sub1A*)—[Flagman/C101Lac × Flagman /Virgo] × [Flagman/A-51 × Khan Dan], IDD—index of blast disease development, Submergence “+”—tolerant to prolonged flooding, Submergence “−”—non-tolerant to prolonged flooding.

## Data Availability

The original contributions presented in this study are included in the article. Further inquiries can be directed to the corresponding author.
